# Joseph C. Wu receives the 2026 ASCI/Stanley J. Korsmeyer Award

**DOI:** 10.1172/JCI209203

**Published:** 2026-06-15

The American Society for Clinical Investigation (ASCI) honors Joseph C. Wu, MD, PhD (Figure 1), with the 2026 Stanley J. Korsmeyer Award. Dr. Wu is recognized for his seminal contributions to the understanding and application of human induced pluripotent stem cells in cardiovascular disease and his impactful mentorship of trainees. He is Director of the Stanford Cardiovascular Institute and the Simon H. Stertzer, MD, Professor of Medicine and Radiology. Dr. Wu was elected to the ASCI in 2012 (Councilor, 2014–2017). He is also a member of the Association of American Physicians (AAP) and National Academy of Medicine, as well as a fellow of the American Association for the Advancement of Science. ASCI President Dr. Priscilla Y. Hsue, Chief of the Division of Cardiology at UCLA, interviewed Dr. Wu at the AAP/ASCI/American Physician Scientists Association Joint Meeting in April 2026.

Priscilla Y. Hsue: Could you please tell us a little bit about yourself, your training path, and how you came to study the problems you’re currently working on in your lab?

Joseph C. Wu: Thank you so much for inviting me. I’ve been an ASCI member for a long time, and I think this is probably the greatest organization to promote the physician-scientist. It’s a great honor to receive this award given how influential Dr. Stanley Korsmeyer was in terms of being a champion for the cause of the physician-scientist.

I did my undergrad at UCLA, and after undergrad, this is back in 1993, I went to Yale for medical school. At that time, I wasn’t sure if I wanted to do an MD versus MD-PhD. I was accepted to the MD-PhD program at UCLA, but I didn’t have the confidence that I could be a physician-scientist, so I went to the MD program instead at Yale. Luckily, at Yale they had a great program in the sense that during medical school you had to do a research thesis. At the same time, they didn’t emphasize grades that much. In fact, I still remember that when you took an exam, you didn’t have to put down your actual name. So every time I took an exam, I put down “Joe Montana.” If I failed, it got posted [as] Joe Montana scored 55. If the cutoff was, say, 70, then [because] it’s an honor system, I needed to report to the teacher. But because of the system, it deemphasized competition among medical students and emphasized doing some type of research. I did my research with Dr. Al Sinusas. At that time, I was interested in being a cardiothoracic surgeon. I did a lot of canine research trying to understand coronary flow dynamics. After four straight summers, I tapped out and said being a cardiothoracic surgeon is very difficult if you want to do research. So [even though] I didn’t go to the MD-PhD program at UCLA, I realized I really enjoy research at Yale because it gave me confidence that I can actually do it.

I published two papers as a medical student. Even though they weren’t in the highest-impact-factor journal, it gave me a lot of joy. So I went back to UCLA for my internship, residency, cardiology fellowship, and I did the STAR program. The STAR program was quite instrumental in that it gave four years of protected time to do research. At that time, the program director was Dr. Joy Frank, followed by Dr. Linda Demer. Dr. Alan Fogelman set up the program, and Dr. Jim Weiss was the chief of cardiology. I would say everybody at UCLA [was] very encouraging of us fellows to go into the program. They gave a lot of guidance and mentorship, and they really believed in the fellows. During that period, I also got quite lucky in picking Dr. Sam Gambhir as my PhD advisor. I learned quite a bit from him during the four years in his lab. We were working on some of the cutting-edge technologies: how do you do molecular imaging to track stem cells, to track gene therapy? Because of my experience in his lab, I felt like finally, finally I could kind of cut it as a physician-scientist. It’s a long-winded way to answer your question. But I must say that along the way, there were a lot of influential figures who guided my decision to stay, to become a physician-scientist.

PYH: How did you first become interested in using stem cell models for cardiovascular disease?

JCW: My PhD was mostly on molecular imaging. Molecular imaging just means that instead of using traditional immunofluorescence in which you have to “sacrifice the animal” to stain the tissue to see where your gene expression is, where your stem cells are, Dr. Gambhir and his colleagues developed techniques in which you could image and see where the stem cells are or where the gene therapy is. He was interested in mostly cancer stem cells, whereas I went to his lab and I said, “Can I use the same technique and track what happened to cardiovascular stem cells?” During my PhD, we were working mostly with adult stem cells, with mesenchymal stem cells, with bone marrow stem cells. Also, we were working quite a bit with cardiac gene therapy, because at that time it was quite hot. People were using adenovirus to overexpress VEGF to see if it could induce angiogenesis in the heart. But what happened was that when we injected these bone marrow cells, the mesenchymal stem cells, or the adenovirus with VEGF into the heart, by imaging we could track within the same animal that most of the stem cells were gone in about two weeks.

Likewise, by imaging, we could track within the same animal that most of the adenoviral VEGF gene expression was at two weeks. I was doing my PhD in pharmacology. And in pharmacology, the first thing they teach you is PK/PD — pharmacokinetics and pharmacodynamics. I realized, “Wait, that doesn’t probably make sense, because if the cells are gone in two weeks, if the genes are gone in two weeks, then it’s probably very difficult to get a durable angiogenesis or durable engraftment.” So when I went to Stanford in 2004, we started playing with other cell types, specifically human embryonic stem (ES) cells. Back then, there was a [federal] ban on using human ES cells. But because I had a starter package from Stanford and the starter package was not federal money, it wasn’t encumbered by the ban. I can still remember that: when you bought equipment, you had to document whether it was NIH funded or not. Because I didn’t have NIH funding, I could buy any equipment I wanted. So we started working with human ES cells. We worked with a couple labs, for example, Dr. Julie Baker’s lab at Stanford. We learned from her lab how you grow and track human ES cells. I still remember one of the early experiments that we did was we labeled human ES cell with firefly luciferase and with GFP and HSV-tk — something called triple fusion reporter gene — and we injected [it] into the heart, and the signal took off: at two weeks, three weeks, four weeks, the signal went up very high. I’d never seen those signals, because as I mentioned earlier, our experience with bone marrow cells, mesenchymal stem cells, was that most of the signals disappeared by about two to three weeks. And when we sacrificed the animal, it turned out to be a teratoma. Teratoma just means that the human ES cell, because it was unguided, became a mixture of different cell types within the body. You could identify bone, brain, liver, kidney, and heart cells. It was disappointing, but at the same time, it opened my eyes. [I] said, “Whoa, this is a cell type that can become any cell type in your body.” Therefore, you should be able to take human ES cells and add different growth factors, different cytokines, different cocktails, so that you could push the human ES cell into brain cells, into heart cells, into liver cells, into kidney cells.

We spent a lot of effort between 2004 and 2006 to take human ES cells and [discover] how to push [them] into beating cardiomyocytes. Around 2006, Dr. Shinya Yamanaka published a paper in *Cell* showing that you could take a mouse fibroblast, add four genes into it, and it will become a mouse iPS cell — induced pluripotent stem cell. These iPS cells for all practical purposes are very similar to the mouse ES cell. These mouse iPS cells can become all the different cell types. He followed that up in 2007 to show that you could do the same thing in human iPS cells, meaning that you could take a human skin fibroblast, add the four genes, and make it a human iPS cell, and then differentiate all the cell types.

It dawned upon me, as a physician-scientist, that the main difference between human ES cells and human iPS cells is that for human ES cells, I never know who the donor was because it’s a “destroyed embryo”; whereas with human iPS cells, I know exactly who that patient is, and I could follow that patient over time, and I can use that patient’s clinical history to correlate it with what happened to the human iPS cells. Essentially, I’m creating a biological twin. So we decided to pivot from human ES cells — and went all in with the human iPS cells. Luckily, we had some early grant funding from the California Institute for Regenerative Medicine. And it was up to our imagination to see what is it that we need to add to push human iPS cells into cardiomyocytes, into fibroblasts, coronary endothelial cells, pericytes, smooth muscle cells — what I call the big five cell types within the heart — as well as other cell types such as macrophages. We spent a lot of time developing these protocols so that we can reliably and reproducibly generate these cells. Once you’re able to generate human cardiomyocytes from healthy [individuals] versus patients with long QT syndrome versus patients with hypertrophic cardiomyopathy, dilated cardiomyopathy, ARVC [arrhythmogenic right ventricular cardiomyopathy], then, again, it’s up to your imagination to see how you’re going to use this for disease modeling.

PYH: I’m a little shocked to hear you say that early in your career, you did not feel confident to pursue [a career as a] physician-scientist. What factors do you feel have been very critical to your success in the lab?

JCW: I come from a humble background. My brother and I were the first kids in the extended family to go to college, and I’m definitely the first one to go to medical school. It’s not like I come from a long tradition of a family of doctors. So I didn’t have confidence in the beginning that I could cut it. But then, as I mentioned earlier, I got lucky in the sense that at Yale, they emphasized research. At UCLA, the STAR program also emphasized research and provided great mentorship. And then I was lucky to be mentored by Dr. Gambhir, who has unfortunately passed away. But the main thing that’s important for the success of any physician-scientist, any trainee, any PhD scientist, clinician-scientist, is mentorship. Finding the right mentor can really make a big difference. There are a lot of talks about finding resiliency and having the right mentor, having the right environment for your research. Certainly, Stanford and UCLA are great environments for research. And that’s part of the reason why I’ve been able to succeed at Stanford.

People talk about networking and about having grit and so forth. I tried to boil down to something very simple for our cardiology fellows or for our postdoc trainees, because I know all of them will probably think in the same way as I was thinking 25 years ago: that is, if I set up a lab or if I go into research, can I do [it] exactly the same way as this big PI or big physician-scientist is doing? I would sit down with the fellow, the postdoc, and I would say — pretend it’s John — “Hey, John, you want to become a physician-scientist, right?” And I just say, “It’s very, very simple. Number one, are you okay with the pay cut? You have to be okay with that because you’re not working as hard as our full-time clinicians.” And John would say, “I’m okay with the pay cut.” Number two, I would ask, “Is your spouse okay with the pay cut?” And these are very important questions, because I do think that a lot of the young MD-PhD students don’t understand these practical questions.

And John says, “I think my girlfriend or my wife is okay.” Number three, I say, “Are you willing to come in on the weekends writing grants and papers?” Because as a physician-scientist — everybody has 24 hours in a day, and so you’ve got to squeeze in time somewhere to come in to write grants and papers. And I think if the answer is yes, yes, yes, then I tell him or her that number four, “Find a great mentor to help guide you, to show you how it’s done. And after that, it’s not that hard.” And so that’s why I try to de-escalate the fear and de-risk everything for the trainee and say, “If you say yes and find a great mentor, it is not that hard to stay in this game to be a physician-scientist.”

PYH: Good advice. How has the preclinical drug discovery process evolved with the integration of human-relevant in vitro models and AI approaches, and what do you see on the horizon?

JCW: I’m a believer that you want to integrate and take advantage of all the new tools that are available to advance the science, because at the end of the day, you want to come up with something to cure and treat and help patients. A lot of these tools that are present, they were not present when I was doing my PhD. In our lab, we do a lot of next-generation sequencing. All the sequencing techniques, the single-cell RNA sequencing, the single-cell ATAC-seq: those were not available back around 2000, 2004, when I was doing my PhD. [Then there is] CRISPR editing, which came around 2010; and iPS cell technology, which came about in 2006, 2007; and last, AI. Individually, these are all very important, powerful techniques, but they’re not able to help you address the question of drug discovery.

You need to combine all four of them to help you accelerate that step. If you take a look at the advances that have occurred in the past 20 years, and if you’re able to say, “For all diseases, if I want to come up with the AI-based drug discovery, I need to start with the disease,” I’m going backward, right? Most of the time, people want to have a technique and then with the technique come up with a cure for disease. That’s what I call A to Z. I’m going from Z to A. Z means I need to start with the disease. What is the unmet need about the disease? Why is it that some treatments work and some treatments don’t work? What is the mechanism of the disease? And then what is the target for that disease? Once I identify the target, then I could use, for example, AI software to figure out what the protein structure is — such as AlphaFold — and also some of the x-ray crystallography structures. I can then use AI software to help me do predictions of what drugs dock to that particular protein domain. And once I synthesize these drugs, I can test them on patient-specific organoids or stem cells that have the disease phenotype. If I want to come up with a drug for Brugada syndrome, a drug for dilated cardiomyopathy, I want to test it in human iPS cell–derived cardio-organoids that have Brugada syndrome, rather than a mouse model, because the phenotype is very different. Then I can then see what drugs work and what drugs don’t work. By integrating functional genomics — to confirm that the target is the culprit — I can then do CRISPR editing on these cells to knock in and knock out the particular gene to show the disease phenotype goes away. Then I can do it on a large scale, because I can go to my clinical-trial-in-a-dish model by recruiting a lot of Brugada patients or a lot of dilated cardiomyopathy patients, have these cells on a dish, test how the drug works and why the drug doesn’t work. You could do a lot of this type of research right now for drug discovery just by using the four models I mentioned: next-generation sequencing, CRISPR editing, iPS cells, and the AI-based platform.

PYH: The next question is about how you’ve approached mentorship in your lab and your role as institute director.

JCW: As an institute director, I interact with many postdocs, many graduate students, and many young and middle-career faculty. In my opinion, the biggest reason we’re in [academia] is to help train the next generation of scientists, the next generation of clinicians, and next generation of physician-scientists. It serves no good if we don’t have the next set of people taking the baton. Because I’m not going to be in this field for the next 50 years. At some time I have to retire, in the next 10, 15 years, and pass it off to the next generation. So it is very important for me personally, and it is also very important that for our Stanford Cardiovascular Institute — we have a great infrastructure set up. For the Cardiovascular Institute, for example, we have six different T32 training grants looking at imaging, vascular biology, myocardial biology, computational science, epidemiology. We have an R38 grant to train physician-scientists. We also have an R25 and a T35 grant by which each year we bring in about 40–50 undergrads throughout the country who are not traditionally exposed to research to Stanford to work with different faculty within the institute for ten weeks to learn how research is done.

They can then go back to their home institution and apply to MD or PhD programs. As the institute director, I write a lot of letters for people who come through this summer program, and they then apply to these graduate schools. A lot of times they’ll email me back and say, “Dear Dr. Wu, I got into this or that medical school.” It gives a lot of satisfaction to see that the trainees who come through this program get into it. I think on an individual-lab level, I also place a lot of emphasis that my trainees do well. For example, I would teach them how to write a grant, review a grant, network, understand what the most important problems in research are, and be able to have a keen acumen and say that this is important, this is not important. Luckily, over the past 20 years, by my last count we’ve had 62 trainees who’ve become PIs and set up their own labs. Last year, for example, we had postdocs who went to Johns Hopkins, WashU, UT Southwestern, The Ohio State University, the University of Montreal, and the University of Galway in Ireland, and last month to UCLA. It’s almost like airplanes taxiing, ready to take off. It’s just a great joy to see the next generation of trainees doing so successfully and then able to take science to the next step. Because ultimately, as I mentioned earlier, all of us want to improve human health. And these are the folks who can help me and help everybody to take it to the next step.

PYH: You touched upon this already, but how do we best encourage and support young physician-scientists in their career?

JCW: I think mentorship is the key. I try to tell young physician-scientists that it is not that hard if you follow those four principles: I mentioned earlier the pay cut; is the spouse okay with the pay cut; working hard on the weekends because running a lab is almost like running a business; then also finding the right mentor. I also advise them that, especially during their training phase, they just need to learn the three principles, at least in my lab. They have to work hard, because you can’t succeed if you’re not working hard. Number two, they have to work smart, meaning that if there are new technologies, incorporate them. If the fundamental hypothesis is incorrect, you have to be able to be nimble enough to pivot to another angle, to another direction. And number three, I tell them that you have to work collaboratively. I always ask them which one of the three is the most important. Sometimes they guess one or the other, but I always emphasize to them that among these three, the most important part is working together, because you can only go so far by working hard, you can only go so far by working smart, but if you work together as a team, you can go much, much, much farther. I hope all my trainees understand that, and I do hope that young physician-scientists understand that working together as a team — because science is a team sport — it is the most important thing: being collaborative, being collegial, willing to share materials, share ideas. Because ultimately, you’re not doing it for your own sake, you’re doing it because you want to advance science to help our patients.

PYH: Thank you so much for taking the time to talk to me today, and a very big congratulations on receiving this Milestone Award.

JCW: Thank you so much.

*The interview has been edited for length and clarity.*

## Figures and Tables

**Figure 1:**
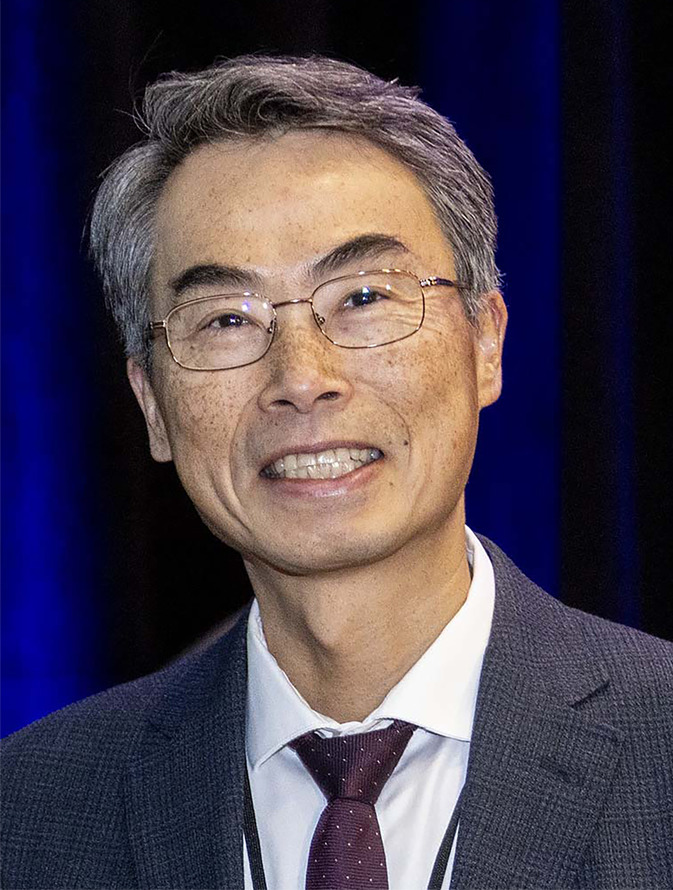
Joseph C. Wu is the recipient of the Stanley J. Korsmeyer Award.

## Version 1 06/15/2026

Electronic publication

